# The Ameliorative Effects of l-2-Oxothiazolidine-4-Carboxylate on Acetaminophen-Induced Hepatotoxicity in Mice

**DOI:** 10.3390/molecules18033467

**Published:** 2013-03-18

**Authors:** Jiwon Choi, Kwang-Hyun Park, Sung Zoo Kim, Jun Ho Shin, Seon-Il Jang

**Affiliations:** 1 Department of Radiological Sciences, Jeonju University, Jeonju 560-759, Korea; E-Mail: jwchoi@jj.ac.kr; 2 Department of Physiology, School of Medicine, Chonbuk National University, Jeonju 561-803, Korea; E-Mail: szkim@jbnu.ac.kr; 3 Department of Oriental Pharmaceutical Development, Nambu University, Gwangju 506-706, Korea; E-Mail: khpark@nambu.ac.kr; 4 Ato Q&A Incorperation, Jeonju 560-759, Korea; E-Mail: maum1675@naver.com; 5 Department of Health & Science, Jeonju University, Jeonju 560-759, Korea

**Keywords:** l-2-oxothiazolidine-4-carboxylate, acetaminophen-induced hepatotoxicity, apoptosis, oxidative stress, antioxidant

## Abstract

The aim of the study was to investigate the ameliorative effects and the mechanism of action of l-2-oxothiazolidine-4-carboxylate (OTC) on acetaminophen (APAP)-induced hepatotoxicity in mice. Mice were randomly divided into six groups: normal control group, APAP only treated group, APAP + 25 mg/kg OTC, APAP + 50 mg/kg OTC, APAP + 100 mg/kg OTC, and APAP + 100 mg/kg *N*-acetylcysteine (NAC) as a reference control group. OTC treatment significantly reduced serum alanine aminotransferase and aspartate aminotransferase levels in a dose dependent manner. OTC treatment was markedly increased glutathione (GSH) production and glutathione peroxidase (GSH-px) activity in a dose dependent manner. The contents of malondialdehyde and 4-hydroxynonenal in liver tissues were significantly decreased by administration of OTC and the inhibitory effect of OTC was similar to that of NAC. Moreover, OTC treatment on APAP-induced hepatotoxicity significantly reduced the formation of nitrotyrosin and terminal deoxynucleotidyl transferase dUTP nick end labeling positive areas of liver tissues in a dose dependent manner. Furthermore, the activity of caspase-3 in liver tissues was reduced by administration of OTC in a dose dependent manner. The ameliorative effects of OTC on APAP-induced liver damage in mice was similar to that of NAC. These results suggest that OTC has ameliorative effects on APAP-induced hepatotoxicity in mice through anti-oxidative stress and anti-apoptotic processes.

## 1. Introduction

Acetaminophen (APAP) is one of the most common analgesic and antipyretic drug and it has been used as a powerful tool to study mechanisms of hepatotoxicity. Although it is safe at therapeutic doses, overdoses of APAP can cause severe liver injury or acute liver failure and during the last decade, APAP indeed became the most frequent cause of acute liver failure in many countries [1]. APAP-induced reactive metabolites deplete glutathione (GSH) and subsequently cause protein binding, as a critical event in the toxicity [2,3,4]. Based on this mechanism, *N*-acetylcysteine (NAC) has been used to treat patients with APAP-induced toxicity [5], and NAC is the most popular therapeutic agent for this application [6]. The effective therapy against APAP overdose-induced hepatotoxicity is GSH replacement in order to scavenge reactive metabolites such as *N*-acetyl-*p*-benzoquinone imine (NAPQI), which is accomplished with NAC and other sulfhydryl donors. However, NAC needs to be given within 12 to 24 h of APAP ingestion. Patients presenting later may benefit from increased metabolic flux, but the likelihood of a positive outcome is notably decreased [7,8].

l-2-oxothiazolidine-4-carboxylate (C_4_H_5_NO_3_S, FW 147.16; OTC) is a prodrug of cysteine. It is a substrate of 5-oxoprolinase, an ubiquitous intracellular enzyme, which generates cysteine from OTC intracellularly [9,10]. OTC exhibits ameliorative properties suggesting a number of potential clinical applications in *in vitro* and *in vivo* experiments [11]. In other studies, NAC and OTC replenished cellular glutathione stores in situations in which glutathione is acutely depleted due to the detoxification of a toxic drug metabolite in animal models [12,13,14]. OTC can also increase intracellular concentrations of GSH above physiological concentrations [10,15,16,17]. One of the major functions of GSH is the detoxification of reactive species and toxic oxygen metabolites generated by the endogenous and exogenous metabolism pathways [18]. Glutathione peroxidase mediates scavenging of intermediates such as hydrogen peroxide and hydroperoxides at the expense of GSH [11]. Therefore modulation of GSH and GSH-px is increasingly important in oxidative stress related diseases and their care. To the best of our knowledge, the action of OTC against APAP-induced liver injury in mice has not been demonstrated systematically. Hence, the present study focused on evaluating the ameliorative effects of OTC against APAP-induced liver damage and its mechanism(s) of action in a murine model.

## 2. Results and Discussion

### 2.1. OTC Treatment was Effective in Preventing APAP-induced Liver Damage

**Figure 1:**
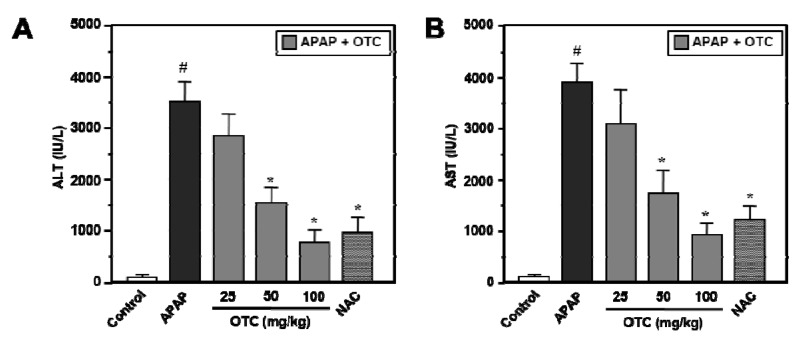
OTC administration ameliorates blood ALT and AST levels in APAP-induced hepatotoxicity model mice. Mice were orally treated with saline or OTC or NAC 2 h before injection of 300 mg/kg APAP. Twelve h after APAP injection, serum was collected for measurement of ALT and AST. (**A**) ALT and (**B**) AST levels were measured in serum of both mice groups. Data represent mean ± SE of n = 6 animals per group. ^#^ *p* < 0.001 *vs.* control group, * *p* < 0.01 *vs.* APAP alone group.

Male BALB/c mice treated with a 300 mg/kg of APAP showed evidence of significant liver damage at 12 h, as indicated by the notable increase of serum alanine aminotransferase (ALT) and aspartate aminotransferase (AST) levels (Figure 1). ALT and AST were significantly increased in the APAP administrated group compared to the normal control group (*p* < 0.001). However, OTC administration (50 mg/kg and 100 mg/kg) significantly reduced serum ALT and AST levels (Figure 1). NAC (100 mg/kg), positive control to APAP-induced liver damage, also show a similar reductive effect towards APAP-induced elevation of serum levels of ALT and AST.

### 2.2. OTC Treatment had Effects on GSH and GSH-Peroxidase Recovery

**Figure 2:**
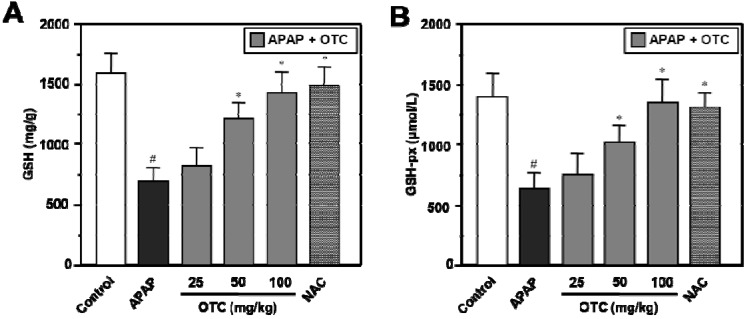
OTC administration elevates GSH and GSH-px levels in APAP-induced hepatotoxicity. Mice were orally treated with saline or OTC or NAC 2 h before injection of 300 mg/kg APAP. Twelve h after APAP injection, liver tissues were collected for measurement of GSH and glutathione peroxidase. GSH (**A**) and glutathione-px (**B**) levels are ameliorated in a dose dependent manner of OTC in APAP-overdose mice. Data represent mean ± SE of n = 6 animals per group. ^#^ *p* < 0.01 *vs*. control group, * *p* < 0.05 *vs*. APAP alone group.

OTC and NAC treatments resulted in a complete recovery of GSH and GSH-px levels in liver tissues (Figure 2). APAP treatment resulted in depletion of liver GSH levels at 12 h, however the liver GSH content was further increased in a dose dependent manner by OTC compared to APAP alone (Figure 2A). Administration of 50 mg/kg and 100 mg/kg OTC significantly elevated the liver GSH levels compared to the APAP only group (*p* < 0.05), however 25 mg/kg OTC produced no relevant recovery of the liver GSH levels. NAC also recovered the liver GSH levels with an effect similar to that of OTC in our APAP-induced liver damage model. OTC administration recovered the GSH-px levels depleted with APAP treatment in a dose dependent manner (Figure 2B). Decrease of the liver GSH-px level were significantly recovered by 50 mg/kg and 100 mg/kg OTC treatment (*p* < 0.05), however 25 mg/kg OTC showed no relevant recovery of the liver GSH and GSH-px level. NAC also recovered the liver GSH and GSH-px level similar to effect of OTC in the APAP-induced liver damage model.

### 2.3. MDA and 4-HNE Level

**Figure 3:**
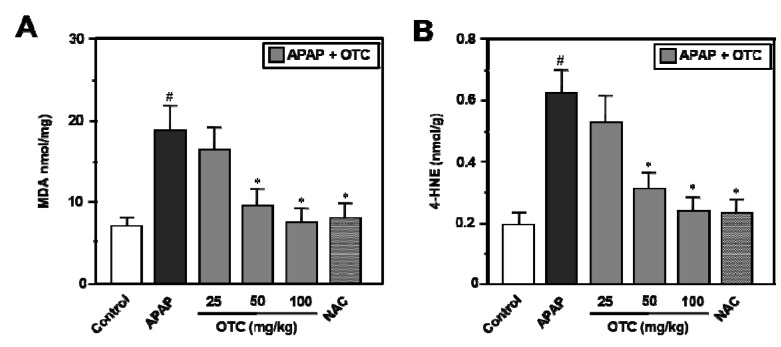
OTC administration reduces MDA and 4-HNE levels in APAP-induced hepatotoxicity. Mice were orally treated with saline or OTC or NAC 2 h before injection of 300 mg/kg APAP. Twelve h after APAP injection, liver tissues were collected for measurement of MDA and 4-HNE. MDA (**A**) and 4-HNE (**B**) levels were decreased in APAP-overdose mice in a dose dependent manner by OTC. Data represent mean ± SE of n = 6 animals per group. ^#^ *p* < 0.005 *vs*. control group, * *p* < 0.01 *vs*. APAP alone group.

**Figure 4:**
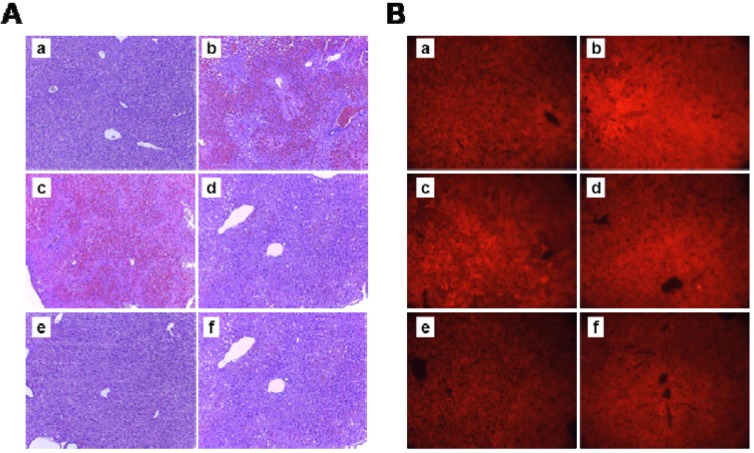
Ameliorative effects of OTC on APAP-induced centrilobular necrosis and nitrotyrosine production. Mice were orally treated with saline or OTC or NAC 2 h before injection of 300 mg/kg APAP. Twelve h after APAP injection, liver sections were stained with (**A**) H&E and (**B**) anti-nitrotyrosin antibody. Representative images of the livers from (**a**) untreated control mice, (**b**) 300 mg/kg APAP, (**c**) APAP + 25 mg/kg OTC, (**d**) APAP + 50 mg/kg OTC, (**e**) APAP + 100 mg/kg OTC, and (**f**) APAP + 100 mg/kg NAC. Original magnification = × 50.

Twelve hours after APAP administration, MDA and 4-HNE levels were increased 2.1- and 3-fold, respectively, in the APAP group compared with the normal group (Figure 3). OTC treatment significantly alleviated APAP-induced MDA (Figure 3A) and 4-HNE (Figure 3B) production in a dose dependent manner. 

In mice receiving 50 mg/kg and 100 mg/kg of OTC in the APAP-induced hepatotoxicity model, MDA levels were significantly reduced compared to the APAP alone group, and levels of 4-HNE were also significantly reduced compared to the APAP alone group.

### 2.4. Histologic Observation and Caspase-3 Activity

Histopathological analysis of the APAP alone treated mice showed severe centrilobular necrosis, lymphocyte infiltration and nitrotyrosine development. The vacuolization, cell swelling and abnormal architecture around the centrilobular veins documents that the cells were undergoing necrosis (Figure 4A) and nitrotyrosine formation was significantly increased in the group administrated APAP (Figure 4B). However, OTC treatment significantly alleviated the APAP-induced liver injuries in a dose dependent manner. NAC also recovered the histological parameters similar to the effect of OTC in the APAP-induced liver damage model.

**Figure 5:**
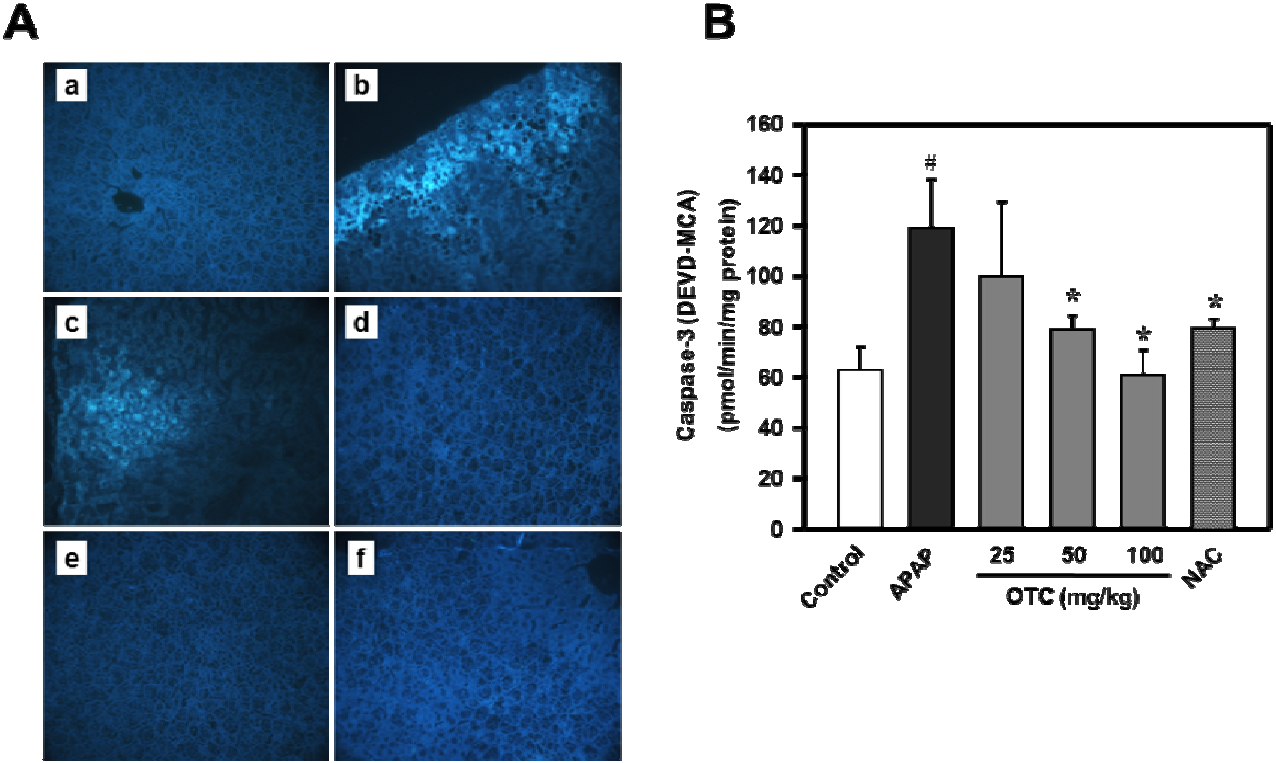
APAP-induced increase of apoptotic bodies and caspase-3 activation were reduced by OTC administration in mice liver. Mice were orally treated with saline or OTC or NAC 2 h before injection of 300 mg/kg APAP. Twelve h after APAP injection (**A**) liver sections were stained with TUNEL reagents. Representative images of livers from (**a**) untreated control mice, (**b**) 300 mg/kg APAP, (**c**) APAP + 25 mg/kg OTC, (**d**) APAP + 50 mg/kg OTC, (**e**) APAP + 100 mg/kg OTC, and (**f**) APAP + 100 mg/kg NAC. Original magnification = ×50, n = 6 of each groups. (**B**) APAP-induced increase of liver casepase-3 activity is decreased by OTC administration. Average caspase-3 activity was measured in tissue lysates (200 μg protein) from APAP-overdose mice. Data represent mean ± SE of n = 6 animals per time point. ^#^ *p* < 0.01 *vs.* control group, * *p* < 0.05 *vs.* APAP alone group.

The extent of necrosis was correlated with massive DNA fragmentation as demonstrated by the TUNEL assay (Figure 5A). Treatment with OTC markedly reduced the area of apoptosis, the number of TUNEL-positive cells, and nitrotyrosine protein adducts. Treatment with NAC also attenuated the areas of apoptosis, the extent of DNA damage and nitrotyrosine staining. Therefore, these results suggest that treatment with OTC after APAP administration was effective in protecting the liver compared to NAC. To determine whether or not this anti-apoptosis effect of OTC could be involved in the APAP-induced liver damage, caspase-3 activity was measured in the liver tissue lysates (Figure 5B). Caspase-3 activity was clearly elevated in the APAP administrated group compared with the no treatment normal group. Administration of OTC significantly reduced caspase-3 activation compared to the APAP only group in a dose dependent manner, however 25 mg/kg OTC showed no relevant recovery of the liver GSH levels. NAC also recovered histological parameters with an effect similar to that of OTC in the APAP-induced liver damage model. These results suggested that APAP-induced liver damage was related to the mechanism of cell death through apoptosis.

### 2.5. Discussion

The goals of the present study were to examine the effectiveness of the l-cysteine pro-drug OTC in its ability to prevent APAP-induced liver damage. In this study, although 25 mg/kg OTC showed no relevant hepatoprotective effects in the APAP-induced hepatotoxicity model mice, above 50 mg/kg OTC had protective effects. OTC administration reduced APAP-induced serum AST and ALT levels and showed recovery of GSH, GSH-px levels in APAP-damaged liver tissue. Administration of OTC decreased centrilobular necrosis, MDA and 4-HNE formations, TUNEL positive area and caspase-3 activation in liver tissues of APAP-induced hepatotoxic model mice.

OTC was synthesized by reaction of l-cysteine with phenyl chloroformate [17]. OTC is useful in intracellular enzyme studies because it is an intracellular delivery system for l-cysteine [9,10,18]. OTC is very soluble and stable in solution, and converted intracellulary to l-cysteine by 5-oxo-l-prolinase via the unstable intermediate, *S*-carboxycysteine [9,19]. Effects of OTC by replenishment of tissue GSH levels were also reported in an endotoxin-induced acutely septic mouse model [20], cardiac dysfunction [21], hepatotoxicity model [22] and asthma [23]. 

OTC administration ameliorated APAP-induced leakage of cellular enzymes into plasma which is a sign of hepatic tissue damage. Measurement of serum ALT and AST levels are used as important diagnostic markers to indicate liver injury due to hepatotoxins [24,25]. Pretreatment with OTC decreased serum ALT and AST levels in APAP-induced hepatotoxicity model mice (Figure 1) and liver damages in the form of changed liver architectures were recovered in a dose dependent manner (Figure 4). 

MDA is a product of polyunsaturated fatty acid (PUFA) peroxidation and is a good marker of lipid peroxidation [26], which is related to APAP-induced tissue damage. MDA levels were significantly increased in plasma [27,28], hepatocytes [29] and liver tissues, and reduced by natural compound extracts. Therefore, administration of OTC broke the chain reaction of lipid peroxidation evidenced, for example, by MDA formation in the APAP-induced hepatotoxicity model. Thus, we suggest that the therapeutic potential of OTC is dependent on an antioxidant mechanism.

4-HNE-protein adducts may be considered a particularly good marker of lipid oxidation during liver injury. Indeed, the demonstrated adduct formation reaction of 4-HNE with important signaling proteins strongly suggests a pathogenic role for lipid aldehyde in the progression of liver diseases [30,31]. In this study, levels of 4-HNE in liver tissue were higher in mice that were given APAP alone, whereas OTC administration reduced 4-HNE adduction in APAP-induced hepatotoxicity model mice. This result suggested that the OTC may be partly involved in 4-HNE metabolism to counteract the APAP-induced hepatotoxicity.

Nitration of tyrosine (*i.e*., formation of nitrotyrosine) has been shown to be an excellent biomarker of peroxynitrite formation [32,33] and it was shown that nitrotyrosine occurs in the centrilobular cells of the liver of acetaminophen-treated mice. Peroxynitrite formation is formed by a rapid reaction between nitric oxide and superoxide, and peroxynitrile production was increased under APAP-toxicity conditions [34]. It is normally detoxified by GSH and GSH px, and GSH is depleted in acetaminophen toxicity [35]. GSH px is a key enzyme in this defense mechanism [36]. In some reports, neither OTC nor *N*-acetylcysteine resulted in statistically significant increases in plasma GSH in normal healthy volunteers at 8 h [14].

In this study, OTC administration significantly reduced nitrotyrosine levels in a dose dependent manner in liver tissues of APAP-induced hepatotoxicity model mice. This result suggest that GSH and GSH px activation is induced by OTC administration and this is consistent with other parameters illustrating the hepatoprotective nature of OTC. The APAP-induced hepatotoxicity serves as an example of the interrelationship between apoptotic and necrotic cell death and their common origin in mitochondrial dysfunction, and overdoses of APAP are a frequent cause of acute drug-induced liver failure [37]. As in other mechanisms of liver failure, the roles played by apoptosis and oncotic necrosis in APAP-induced liver failure have been controversial. Caspase-3 protein is a member of the cysteine-aspartic acid protease (caspase) family [38] and caspase-3 shares many of the typical characteristics common to all currently-known caspases [39]. Acetaminophen triggered the release of cytochrome c from mitochondria into the cytosol, activation of caspase-3, 8, and 9, cleavage of poly(ADP-ribose) polymerase, and degradation of lamin B1 and DNA [40]. Several reports have shown that APAP-induced liver apoptosis was inhibited by repression of mitochondrial apoptotic signaling [41], preventing down-regulation of Bcl-2 and up-regulation of Bax [42], regulation of connexin [43], and cleaved caspase-3 expression [43] caused by maintaining of hepatic glutathione homeostasis [44]. In present study, we observed APAP-induced apoptotic signaling with TUNEL staining of liver tissue and cleavage of caspase-3, and this was significantly reduced by OTC administration. Therefore, these results suggested that OTC is an effective substance against liver injuries. 

## 3. Experimental

### 3.1. Materials

APAP, OTC and other chemicals were purchased from Sigma-Aldrich (St. Louis, MO, USA). ALT and AST levels in serum were measured using ALT and AST assay kits (ASAN Pharmaceutical. Seoul, Korea). GSH and GSH-px assay kits were obtained from United States Biological (Salem, MA, USA). Enzyme linked immunosorbent assay (ELISA) kits for MDA and 4-HNE were purchased from Cell Biolabs, Inc. (San Diego, CA, USA). Caspase-3 activity and TUNEL staining kits were purchased from R&D Systems (Minneapolis, MN, USA). Specific antibody against nitrotyrosin for immunohistochemistry was obtained from Specific antibody against nitrotyrosin for immunohistochemistry was obtained from Santa Cruz Biotechnology (Santa Cruz, CA, USA). All assay kits were used according to the corresponding manufacturer’s instructions.

### 3.2. Animals

Male BALB/c mice were purchased from The Orient Bio (SungNam, Korea) and housed in an environmentally controlled room with a 12-hour light and dark cycle and *ad libitum* access to food and water. Mice were starved overnight and orally administrated OTC or NAC. After 2 h, 300 mg/kg APAP in warm saline was administered in a single i.p. injection and then animals were sacrificed by cervical dislocation 12 h later. Blood was collected from the inferior vena cava, placed in clean Eppendorf tubes for 3 h at room temperature, and then the clotted blood was centrifuged at 14,000–20,000 × g for 15–20 min at 4 °C. Livers were excised, and small sections were fixed in 10% phosphate-buffered formalin for histological analysis. 

### 3.3. Caspase-3 Activity Assay

The caspase-3 activity assay was performed using a colorimetric activity assay kit according to the manufacturer’s instructions. In brief, assays were performed by incubating protein from tissue lysate (200 μg) in reaction buffer (100 μL) containing caspase-3 substrate (4 mM DEVD-pNA. 5 μL) in 96-well plates. The reaction buffer contained 1% NP-40, 20 mM Tris-HCl (pH 7.5), 137 mM *N*-acetyl-cysteine, and 10% glycerol. Lysates were incubated at 37 °C for 2 h. Samples were incubated in the dark and measured with a microplate reader (Molecular Devices, Sunnyvale, CA, USA) at an absorbance of 405 nm.

### 3.4. Histology and Immunohistochemistry

Mice were anesthetized with diethyl ether and the liver was incised. Livers were then removed from the mice and fixed overnight in a cold 10% formalin solution. Fixed tissues were processed routinely for paraffin embedding, and 5 μm sections were used for hematoxylin-eosin staining. Stained morphology was analyzed using a microscope (Leica, Wetzlar, Germany). For immunohistochemistry, tissue sections are deparaffinized and stained with anti-nitrotyrosine antibody and TUNEL reagent. The sections were reacted overnight at 4 °C or for the time/temperature indicated in the manufacturer’s instruction, rinsed with PBS and then covered with cover-slips. They were examined by fluorescence microscopy (Olympus, Tokyo, Japan) to assess the molecular expressions or distributions.

### 3.5. Statistical Analysis

Differences in data among the groups were analyzed by one-way ANOVA, and all values were expressed as mean ± S.E.M. The differences between groups were considered to be significant at *p* < 0.05.

## 4. Conclusions

In summary, this study demonstrates the ameliorative effects of OTC administration in APAP-induced hepatotoxicity model mice. Serum AST/ALT levels were reduced by OTC administration and recovery of GSH, GSH-px in liver tissue was shown. Administration of OTC decreased centrilobular necrosis, MDA/4-HNE formations, TUNEL positive area and caspase-3 activation in liver tissues of APAP-induced hepatotoxic model mice. These results suggest that OTC has a potential use for treatment against APAP-induced hepatotoxiccity related diseases.
